# Multilevel selection on individual and group social behaviour in the wild

**DOI:** 10.1098/rspb.2024.3061

**Published:** 2025-03-19

**Authors:** Conner S. Philson, Julien G. A. Martin, Daniel T. Blumstein

**Affiliations:** ^1^ Department of Ecology & Evolutionary Biology, UCLA, Los Angeles, CA 90095, USA; ^2^ Rocky Mountain Biological Laboratory, Crested Butte, CO 81224, USA; ^3^ Natural Reserve System, UCSB, Santa Barbara, CA 93106, USA; ^4^ Department of Biology, University of Ottawa, Ottawa, Ontario, K1N 9A4, Canada

**Keywords:** multilevel selection, social behaviour, social networks, individual social position, group social structure

## Abstract

How phenotypes are shaped by multilevel selection—the theoretical framework proposing natural selection occurs at more than one level of biological organization—is a classic debate in biology. Though social behaviours are a common theoretical example for multilevel selection, it is unknown if and how multilevel selection acts on sociality in the wild. We studied the relative strength of multilevel selection on both individual behaviour and group social structure, quantified with social networks and 19 years of data from a wild, free-living mammal, the yellow-bellied marmot (*Marmota flaviventer*). Contextual analysis (exploring the impact of individual and group social phenotypes on individual fitness, relative to each other) revealed multilevel selection gradients in specific fitness and life history contexts, with selection for group social structure being just as strong, if not stronger, than individual social behaviour. We also found antagonistic multilevel selection gradients within and between levels, potentially explaining why increased sociality is not as beneficial or heritable in this system compared with other social taxa. Thus, the evolutionary dynamics of hierarchical or nested biological traits should be assessed at multiple levels simultaneously to tell a more accurate and comprehensive story. Overall, we provide empirical evidence suggesting that multilevel selection acts on social relationships and structures in the wild and provide direct evidence for a classic, unanswered question in biology.

## 1. Introduction

The evolution of sociality is a central question in biology [1–3]. Classic explanations for the emergence of social group living focus on the individual fitness costs and benefits incurred via, for example, increased resource competition or predator detection and avoidance [1–3]. This has helped develop a general theory for the selection pressures of social group living. However, within social groups, individuals vary in their pattern of social interactions with others and this individual variation, in part, drives differences in emergent group social structure [2,4–8]. In turn, group structures feed back to influence patterns of individual social interactions [7,8].

Despite these two levels of social organization being nested and thus non-independent, both can be quantified as discrete phenotypic levels of biological organization [2,5–9]. These individual and group social phenotypes both have fitness and population dynamic consequences in wild populations [5,6,10–15] and affect health and human ageing [12,16]. While the drivers and consequences of individual social behaviour and group social structure have been explored independently, if and how these two nested social phenotypes are under selection relative to each other remain largely unknown.

Multilevel selection is a theoretical framework positing natural selection simultaneously occurs at multiple levels of biological organization, and at levels other than only the gene [17–22]. The theory of multilevel selection has undergone many transformations over the years through healthy debate [17–19,21,23–26] and has been suggested as a driving force in the emergence of multicellular organisms [25,27], human cultural evolution [22] and the structure of entire ecosystems [28,29].

The modern multilevel selection argument proposes that individual fitness may be impacted by an individual’s phenotype and by the group’s phenotype simultaneously [9,21,25,30]. This modern argument is not suggesting groups have fitness and that there is variation among groups (e.g. as done with the Price–Hamilton approach [31,32]). Instead, individuals are the result of selection on both individual and group phenotypes, as observed in a variety of morphological phenotypes in plants [33] and animals [34–36]. For example, a flower’s fitness may be impacted by how tall its stem is but also impacted by how tall the stems around it are—so both the flower’s individual phenotype and the phenotype of its group impact the individual flower’s fitness. In the context of social behaviour, individual and group social phenotypes also both have documented fitness consequences for individuals too, when either level is explored independently, as seen in some birds [10], fishes [37], insects [9] and mammals [13–15]. Because individuals are impacted by both their individual and their group’s phenotype, a multilevel selection framework can tease apart what selection is on the individual phenotype and what selection is on the group phenotype.

Furthermore, the individual fitness consequences between the two phenotypic levels, when explored independently or simultaneously, do not always align [9,15]. Because the levels do not always align and because the levels feed back to influence each other, natural selection at one level will likely affect the other (either directly or indirectly [38,39]). Selection at one level may increase or inhibit selection at the other, and thus have a stronger impact on the overall evolutionary response [39]. Furthermore, when exploring selection on either level independently, selection on a higher or lower level may be detected at the level being explored, leading to inaccurate or biased evolutionary predictions or false positives. Alternatively, no selection may be detected despite selection occurring at a level not being explored, leading to false negatives and inaccurate evolutionary expectations.

Therefore, for nested traits like social behaviour, selection should not be quantified at either level independently but should be quantified simultaneously at all relevant levels to partition selection among the nested levels. Thus, multilevel selection may be an important mechanism for the evolution and maintenance of the nested individual social behaviours and group social structures that emerge from group living.

Despite social behaviour being a common theoretical example in making the case for multilevel selection [17–19,21–26], prior work has not explored the presence and strength of multilevel selection for social behaviours in wild, free-living populations. Thus, the decades-old debate of individual versus group selection for social behaviours [40], and more recently multilevel selection [20,26], remains largely theoretical and not empirical [25]. If multilevel selection acts on sociality in the wild is an open question in evolutionary biology that has the potential to inform our understanding of the evolutionary origins of social relationships and structures that underpin social lives across species.

To address if multilevel selection acts on sociality in the wild, we used 19 years of continuous social, fitness and life history data on a well studied, wild, free-living population of yellow-bellied marmots (*Marmota flaviventer*). A harem polygynous rodent, yellow-bellied marmots live in colonies and socially interact in summer before hibernating over winter. The evolutionary origins of yellow-bellied marmot group living may be attributable to predator avoidance and/or reflect limited opportunities to disperse to establish new colonies [41]. Marmot social groups are composed of mostly kin, with occasional non-kin immigration (most often a yearling or adult male [41]). Individual social behaviour has a genetic basis [42] and individual behaviour and group structure are environmentally mediated [41–45]. Individuals also experience fitness consequences based on both their individual and group social phenotypes [13–15,46–50], namely, more social individuals have increased summer survival owing to antipredator benefits [50] and are more philopatric potentially because of the costs of dispersal [46], but have decreased hibernation survival (48), reproductive success [47] and longevity [49] potentially owing to the time and energetic costs of social interactions. Individuals residing in more socially connected groups also experience decreased individual reproductive success [14], again owing to energetic costs, but increased individual winter survival [15] as a result of potential social hibernation and thermoregulatory benefits. Increased sociality being largely associated with individual fitness costs is largely unique for social mammals [12], but nevertheless shows that sociality has consequences in this species.

While exploring nested levels of organization independently may lead to inaccurate estimation of selection, these studies exploring the levels of sociality independently provide a strong background to ask informed questions about the importance of multilevel selection for social traits. Thus, with identified genetic and environmental drivers of social phenotypic variance and varying fitness consequences for individuals (when explored independently), this system provides an ideal opportunity to estimate multilevel selection for individual and group social phenotypes in the wild to more accurately partition selection among the nested levels of organization and ultimately obtain a better understanding of the evolution of sociality.

To quantify the social phenotype, we used affiliative social interactions from 172 social groups comprising 723 unique individuals across 19 years to construct social networks [4–6] and calculated a pair of analogous individual and group social network measures for four core social traits (table 1, [9]). Each measure is commonly used across studies in human and non-human animal social network studies [4–6]. The analogous pairs are not strictly required to assess multilevel selection, but because multilevel selection could occur between the social traits, this approach allows clear evaluation of the relative importance of both individual- and group-level selection for specific social traits. To evaluate the distinct contributions of individual social behaviour and group social structure to four annual individual fitness correlates (summer survival, hibernation survival, if a female weaned offspring, and how many offspring a mother had), we used a contextual analysis that uses a partial regression to partition selection among levels [9,30,51]. Contextual analysis defines individual-level selection as the impact that the individual phenotype has on individual fitness and defines group-level selection as the impact that group phenotype has on individual fitness. Despite the inherent non-independence of the individual and group social phenotypes, contextual analysis partitions among the two phenotypic levels relative to each other.

**Table 1.** The four social traits and corresponding analogous pair of individual and group-level social network measures are used to quantify the individual and group social phenotypes.

social trait	individual social trait	group social trait
connectivity	degree: number of social relationships	density: proportion of possible social relationships that are observed
closeness	closeness: number of social links to access all other individuals in the group	inverse average path length^a^: mean social distance between all individuals in the group
breakability	embeddedness: connectedness in their cluster and group	inverse cut points^a^: number of social relationships that if broken/lost result in two or more separate social groups
clustering	clustering coefficient: proportion of an individual’s direct social partners that also interact (i.e. local transitivity)	transitivity: proportion of connected triads actualized

^a^ Inverse values of cut points and average path length were used so that increase in any of the eight variables reflected an increase in sociality.

Because individual social behaviour and group social structure are nested, if we find selection was only observed at one phenotypic level, we could interpret that level as under direct selection while interpreting the other level as under indirect selection [30]. This would not support the presence of direct multilevel selection for social behaviour. If direct selection was observed for both social phenotypes, even across different social traits or contexts, we would have suggestive evidence of multilevel selection for sociality in the wild. Because, when exploring the individual and group levels independently, more social individual and group social phenotypes are mostly costly for individual fitness in this system [13–15,46–48,50], we predicted negative selection (i.e. selection for less sociality) for the connectivity, closeness and clustering social traits at both levels. We predicted positive selection (i.e. selection for being more social) for breakability traits at both phenotypic levels given more socially embedded individuals and less breakable groups (into two or more social groups) have some individual fitness benefits (philopatry [46], hibernation survival [15]). Lastly, we predicted the strength of selection for individual social behaviour would be stronger than selection for group social structure given the former has more known fitness consequences in this system (when explored independently) and because of the common critique of multilevel selection that the strength of selection for individuals is stronger than for groups [24,52].

## 2. Material and methods

### (a) Study system

We used a 19 year dataset (2003−2022) on wild, free-living yellow-bellied marmots (*M. flaviventer*) studied at and around the Rocky Mountain Biological Laboratory in Colorado (38°57’ N, 106°59‘ W; *ca* 2895 m above sea level). Yellow-bellied marmots are hibernating, harem polygynous, facultatively social ground-dwelling squirrels with matrilineal colony structures. It is important to note that these marmots are not cooperative breeders. This population is active for around 5 months annually (mid-April to mid-September). Mating soon after emergence from hibernation, new pups emerge, and yearlings disperse in late June to early July. Annually, most males and about half of females disperse as yearlings, typically resulting in movement out of our study area [41]. Marmots were studied annually at seven colony sites spread across 5 km at the bottom of the valley. Colonies are grouped into higher and lower elevation sites (four are at higher and three are at lower elevation sites). Higher elevation sites are approximately 166 m higher and experience harsher weather conditions [43,44,53].

Throughout the active season, marmots were repeatedly live-trapped, and their social behaviour was systematically quantified from 2003 to 2021. Recapture rate is above 86% in all colonies for all sex and age classes considered [54]. In addition to unique ear tags, all individuals were marked with unique non-toxic dye marks on their dorsal pelage to allow accurate identification. Marmots were weighed when trapped and these data were used to predict 1 June and 15 August body mass (to estimate early and late season body condition) via a best linear unbiased predictions (BLUPs) model [55,56]. June mass reflects the energy trade-off between leftover energy from hibernation and available energy for spring reproduction, whereas August mass reflects gain in fat mass during the active season and predicts overwinter survival [41,55,56]. Data used in our BLUPs consisted of 25 979 observations across 4330 individuals and 58 years. Using BLUPs can lead to higher rates of Type 1 error mainly owing to a decrease in the effect-associated standard error and not an increase in the absolute value of the effect [57,58]. Martin & Pelletier [59] showed that BLUPs can make accurate body mass predictions when there are on average greater than three measurements of body mass per individual. Our data have a mean of 5.99 observations per individual (range: 1.0−107.0; median = 4.0). Mixed with our relatively large dataset used to produce the BLUP [60] and previous sociality research in this system using this BLUP [13,15,44], these factors facilitate the accuracy and reliability of our body mass BLUP. Importantly, mass is included in the model to correct for potential confounding effects and is not a part of the hypothesis of sociality and multilevel selection. The estimate associated with mass should be unbiased using BLUPs adequately to correct for the mass effect; however, the associated probability should be interpreted with care.

### (b) Social networks

Detailed behavioural observational methodology and the ethogram are outlined in Blumstein *et al.* [46]. For social interactions, the initiator, recipient, location, time and type of each interaction were recorded, with most interactions (79%) occurring between identified individuals. The remaining 21% of interactions could not be identified because of the interacting individuals’ posture or visual obstructions. We excluded these interactions from our data, which should not significantly influence our estimates of social structure [61,62]. We only included adults and yearlings because only these cohorts were present in spring, when social interactions were most common, and because pups emerge mid-season and primarily interact with their mother and each other [63]. We eliminated transients by excluding individuals observed or trapped fewer than five times in a given year [47–49,64]. Only interactions in April, May and June (*ca* 2.5 months time frame is when marmots emerge from hibernation/mate to when pups emerge from natal burrows) were used because this is when most social interactions occur and when we have the highest resolution of observation data (the growth of vegetation begins to impair observations as the summer progresses). Lastly, we focused on affiliative interactions (e.g. allogrooming, greeting, play) because they relate to fitness on both the individual and group levels [13–15,47–50] and affiliative interactions constituted 88% of all social interactions.

Marmots share space with a subset of all possible individuals within their colony area. We therefore defined social groups based on space-use overlap per year (two individuals observed using the same burrow or seen/trapped at the same location and time within 1 day intervals). Simple-ratio pairwise association indices based on colony space-use overlap [65] were calculated with SOCPROG (version 2.9; [66]) and run through the random walk algorithm MapEquation [67–69] to assemble association indices and identify social groups (network isolates within an association index). While MapEquation assigns each individual to only one social group (per year in our case), this can exclude key social connections, such as those with adult males. Because adult males often mate with females from multiple matrilines and have important interactions with members of multiple groups, we added adult males to each social group for which they had at least one social interaction with a member of that group to enable more accurate social network measures. However, each year, a male’s network measures and fitness were only calculated from his originally assigned group.

From these spatially defined groups, directed and weighted social interaction matrices were constructed from affiliative social interactions for each group each year with the R (version 4.2.0; [70]) package ‘igraph’ (version 1.4.2 [67]). These affiliative social interaction matrices consisted of 42 369 social interactions between 1294 individuals (338 of whom were observed across multiple years). This operationalization produced 180 social groups with group sizes ranging from 2 to 35 individuals with a mean of 7.65 ± 5.92 (mean ± s.d.). Individuals had an average of 66.23 ± 90.72 social interactions per year, ranging from 1 to 694. Within social groups, social interactions averaged 447.35 ± 653.18, ranging from 2 to 4118.

**Figure 1.**
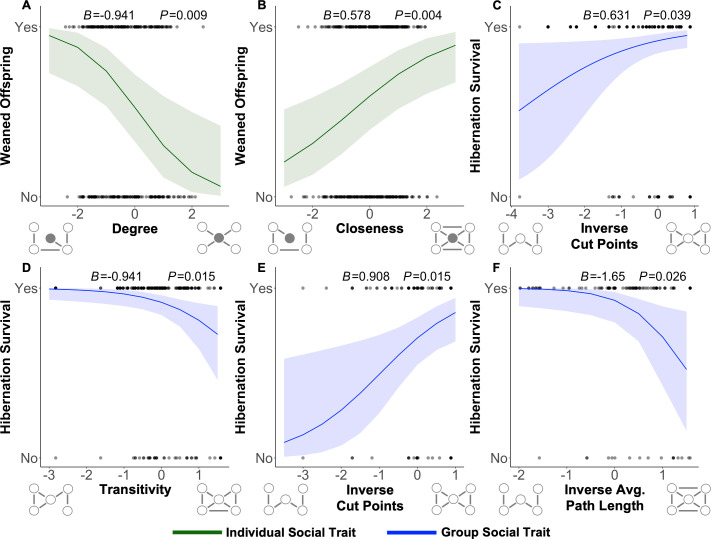
Selection gradients (plotted as marginal effects) for social traits representing the individual and group social phenotypes. (A.B) For the individual social phenotype (green), connectivity was under negative selection (A; fewer social partners) and closeness was under positive selection (B; less social distance from others) for adult female reproductive success. C,D) For the group social phenotype (blue), adult females were under positive selection for breakability (C; more social ties required to break to split into two or more groups) and negative selection for clustering (D; lower proportion of connected transitive loop) for hibernation survival. (E) Group breakability was under positive selection for yearling male hibernation survival. (F) The group closeness trait (mean social distance between all individuals) was under negative selection for adult male hibernation survival. *B* represents the estimate, or strength of selection, and *P* represents the p-value.

We calculated (using ‘igraph’) four pairs of analogous individual and group social network measures to quantify the independent contributions of the individual and group phenotypes (table 1). These analogous network measure pairs quantify four core traits in human and non-human animal social networks (including yellow-bellied marmots) at the two levels of social network organization: connectivity, closeness, breakability and clustering [4,71. Degree (how many social partners an individual has [4]) and density (proportion of possible social connections that are observed in a group [72]) are paired to quantify social connectivity. Closeness (social distance between all other individuals in the group [4]) and average path length (mean social distance between all individuals in the group [72]) quantify social closeness. Embeddedness (individual connectedness based on their direct and indirect relationships with their cluster and group [73]) and cut points (number of social links that if broken or lost result in two or more social groups of at least two individuals [74]) quantify social breakability. Local clustering coefficient (proportion of an individual’s direct social partners that are also social partners; i.e. local transitivity [75]) and transitivity (proportion of possible social triads that are observed in a group; i.e. global transitivity [76]) quantify social clustering. After scaling (see §2d), we flipped the sign for average path length and cut points, so all social network measures’ values correspond to increased sociality. This was done to facilitate interpretation and presentation as the directional slope in figure 1 can be interpreted as the direction of selection for all measures. We thus refer to inverse average path length and inverse cut points throughout.

The reliability of the social network measures is facilitated by our regular observations of marmot social groups (mean *n* observations per individual across years = 28.81, range of each year = 6.79–75.14) and low rate of unknown individuals involved in social interactions [61,62,77]. Social group size (measured as social network size) is associated with many group-level social network measures ([4]; e.g. density, cut points) that may mediate the strength of selection. Because density, inverse average path length and transitivity are already ‘standardized’ based on their equations, we manually standardized inverse cut points by social group size so that all four group-level measures account for social group size in their calculation [4]. This facilitates comparison between our group-level measures and with previous research in this system [13,14].

### (c) Fitness measures

Summer survival was defined as when individuals were seen or trapped after 1 August or in subsequent years, and over-winter/hibernation survival was defined as those individuals having survived the summer being seen the following spring or in subsequent years [15]. For summer survival, only adults (>2 years old) were included in the analysis owing to uncertainty quantifying survival for yearlings because a majority dispersed [78]. Predation accounts for 98% of summer mortality [79], and poor body condition and winter snowpack are primary predictors of hibernation mortality [53,80]. Summer survival was paired with network measures from the current active season, and hibernation survival with network measures from the active season before winter.

Only female reproductive success is quantified because male reproductive success mostly depends on dominance, body condition and tenure length [81,82], which are difficult to quantify, and the smaller number of males in the population diminishes analysis power. We quantified two attributes of female reproductive success: (1) if a female successfully weaned offspring from the burrow, and (2) the number of offspring a mother weaned (if at least one pup was weaned) [14]. Behavioural observations and a comprehensive genetic pedigree [83,84] were used to assign offspring to mothers. This method does not account for pups that may have been born in the burrow but died before emergence (all pups are born in the burrow and emerge *ca* 30 days after birth [41]).

### (d) Contextual analysis

We used contextual analysis, an extension of the Lande–Arnold selection analysis using partial regression to partition selection among levels (in this case, the individual and group social phenotypes; [30,51]). Contextual analysis is generalizable to multiple levels of organization provided that the lowest level included is the level at which fitness variation is being explored. Our contextual analysis differed from classic contextual analysis [30,38,85] since we used emergent group traits instead of using the mean of all individuals within a group.

Contextual analysis scales the phenotypic levels being explored because natural selection is relative, and thus it is the distribution of values, not the absolute values, of a trait that may reveal multilevel selection. Contextual analysis is sensitive to the scale of standardization and should be based on the biological and ecological processes that generate selection in the context of the study system [9,86]. Thus, we mean-variance standardized individual-level social network measures at the scale of each social group within each year (i.e. intra-group). Because group-level selection inherently operates across groups, we standardized group-level social network measures across all social groups across all 19 years (i.e. inter-group). We further mean-variance standardized group size on the global scale across all social groups across all years. Overall, the model can be expressed as:


$$w_{jkl}=\beta_{0}+\beta_{w,\Delta P_{i}}\Delta \;P_{i_{jkl}}+\beta_{w,\Delta P_{g}}\Delta \;P_{g_{jkl}}+e_{jkl},$$

where *w_jkl_* is the relative fitness of individual *j* in group *k* in year *l*, *P_i_* are the social traits of an individual (individual social phenotype) and *P_g_* are the social traits of the social group (group social phenotype). $\Delta P_{i_{jkl}}$ is the deviation of social trait for individual *j* from the mean of its group *k* in year *l*. $\Delta P_{g_{jkl}}$ is the deviation of the social group trait for group *k* of individual *j* in year *l* from the overall mean of social group trait across all groups and all years. $\beta_{w,\Delta P_{i}}$ and $\beta_{w,\Delta P_{g}}$ are the among-individual and among-group selection gradients. $\beta_{0}$ and *e_jkl_* are the intercept and residual terms, respectively [35,51].

Owing to fundamental differences in life history strategies between age classes (adult and yearling) and sexes in this system [41], we fitted separate models for each cohort. Each model included the fitness measure as the response variable, the four pairs of analogous social network traits, social group size and valley location (higher or lower elevation) as fixed effects to account for known environmental and social variation. Individual ID and year were fitted as random effects to further account for environmental and demographic variation. Eight models were fitted in total, and the final models, after correcting fit for multicollinearity, met their respective assumptions. In R (version 4.2.0 [70]), models were fitted with ‘lme4’ (version 1.1-33 [87]) and assumptions were checked with the packages ‘car’ (version 3.1-2; [88,89]) and ‘DHARMa’ (version 0.4.6; [90]).

The set of models (adult females only) for both measures of reproductive success additionally included June body mass as a fixed effect given body condition’s importance for reproductive success in this system [41]; neither model had multicollinearity issues. The model for whether or not a female weaned a litter was fitted with a binomial distribution with a bobyqa optimizer of 10 000 function evaluations and had 363 observations across 157 unique individuals. The model for the number of offspring (if a litter was weaned) was fitted with a Poisson distribution and had 191 observations across 98 unique individuals (electronic supplementary material, table S1).

The four sets of models (one for each age–sex cohort) for hibernation survival additionally included August body mass as a fixed effect [41]. These models were fitted with a binomial distribution and a bobyqa optimizer of 10 000 function evaluations. Multicollinearity was an issue between the network traits and thus the degree–density analogous pair was removed from all four models. With 19 years of data, the yearling male and female models had 119 and 209 observations, respectively (electronic supplementary material, table S2). The adult male model had 109 observations across 58 unique individuals and the adult female model had 324 observations across 134 unique individuals across 19 years (electronic supplementary material, table S3).

The two models for summer survival (male and female adults only) additionally included June body mass and a predation index as fixed effects [41]. The predation index was calculated by dividing the number of predators seen in a colony by the time spent observing that colony for that year [91]. These models were fitted with a binomial distribution and a bobyqa optimizer of 10 000 function evaluations. Both of these models had variance inflation factor (VIF) >5 for density. While contextual analysis accounts for the inherent non-independence of the individual and group social phenotypes by partitioning among the two phenotypic levels relative to each other, we used extra caution and took a more conservative approach by removing the degree–density analogous pair and running the models again. The summer model included 19 years of data with 138 observations across 80 unique individuals for males, and for females had 363 observations across 157 unique individuals (electronic supplementary material, table S4).

Because of the standardization approach we used as part of this contextual analysis, model estimates represent the magnitude of the selection gradient. We also controlled for multiple comparisons by calculating false discovery rate (FDR)-adjusted *p*-values [92–94] based on eight comparisons for the eight models, which still showed multilevel selection occurred (despite losing statistical significance for some group measures after the FDR adjustment; electronic supplementary material, table S5).

In summary, our contextual analysis (i) accounts for the inherent non-independence of the individual and group social phenotypes by partitioning among the two phenotypic levels relative to each other, and (ii) controls for other biologically relevant variables that, if excluded, could otherwise cause spurious associations. If we find significant evidence of selection, it is within the context of the biology of this system, not in isolation. Our contextual analysis permits the quantification of independent contributions to individual fitness at different social phenotypic levels, and between age–sex cohorts, while accounting for natural variation in environmental and demographic factors across a nearly two-decade dataset of a wild, free-living mammal.

## 3. Results and discussion

### (a) Context-dependent multilevel selection

Our results revealed quantitative differences in the presence, strength and direction of multilevel selection for individual and group social phenotypes dependent on sex, age and fitness context (figure 1).

Individual connectivity was under negative selection (i.e. fewer direct social partners) and individual closeness was under positive selection (i.e. socially closer to all others in the group, directly and indirectly) for reproductive success in adult females (figure 1A,B; electronic supplementary material, table S1). This could represent an evolutionary balance in this system between the time and energetic costs of social relationships for reproductive success [47], while still maintaining closer social distance to others to maximize anti-predator benefits (e.g. hearing alarm calls), allowing more time and resources to be devoted to reproduction [50]. Given each species’ time budget and selective pressures are unique, what individual social traits are under selection, and in what way, will be context-dependent based on the biology of that system. For example, in more gregarious species, more aligned selection across individual social traits may be observed.

Group clustering was under negative selection (i.e. there were fewer connected transitive loops) for adult female hibernation survival (figure 1D; electronic supplementary material, table S3), showing selection for adult females residing in less socially connected and clustered social groups. Group closeness was also under negative selection (i.e. larger social distance between all individuals) for adult male hibernation survival (figure 1F; electronic supplementary material, table S3). This shows selection for adult males residing in groups where all individuals are more socially distant. Yellow-bellied marmots are facultatively social, meaning that, compared with more gregarious species, marmots are more socially flexible and sometimes solitary [41]. Thus, marmots may not benefit from residing in more connected social groups, as supported by this negative selection for social structures that may facilitate increased rates of social interactions. However, group breakability was under positive selection (i.e. more social ties required to break a group into two or more separate groups) for both adult female (figure 1C) and yearling male (figure 1E) hibernation survival (electronic supplementary material, tables S2 and S3). That is, selection favours individuals residing in more structurally cohesive and less fragmentable social groups because these groups may facilitate synchrony in hibernation onset [95] and because the breaking of social groups may reduce the number of individuals engaging in social hibernation, reducing subsequent thermoregulatory benefits and increasing the risk of death in hibernation (as seen in the alpine marmot (*Marmota marmota*) [95]). Collectively, this suggests group structures that facilitate phenological timing and thermoregulatory benefits are under positive selection, while structures that foster increased rates of interaction are under negative selection. The particular type of group social structure matters.

Compared with exploring the nested levels of social organization independently, which may lead to an inaccurate estimation of selection, this multilevel selection analysis revealed a more comprehensive picture of selection for sociality in this system, namely only the two group-level breakability (i.e. inverse cut points) results were found previously [15]. This difference could be attributable to multiple factors, such as different-sized datasets and statistical approaches across the studies; the individual social phenotype and reproductive success analysis was conducted over a decade ago [47]. Now with a 19 year dataset and a standardized statistical approach, we have a more accurate estimate of selection for sociality in this system. While exploring the levels of sociality independently provided a strong background to ask informed questions about the importance of multilevel selection for social traits, this multilevel selection approach, partitioning selection among the nested levels of organization, reveals a more informed and representative view of natural selection in this system, facilitating more accurate evolutionary predictions about sociality in this system.

Results somewhat align with our *a priori* predictions of negative selection for less connected, close and clustered social traits at both phenotypic levels and positive selection for less breakable traits at both phenotypic levels. However, individual closeness was under positive selection, contrary to our prediction. Neither social phenotypic level was under selection for summer survival (electronic supplementary material, table S4) or in female yearlings (electronic supplementary material, table S2), also contrary to our *a priori* predictions. Because the two social phenotypic levels are scaled, model estimates represent the magnitude of the selection gradient. Across all traits, age classes, and both sexes, the individual social phenotype had a mean selection gradient of 0.76 (0.26 s.e.) and the group social phenotype a mean of 1.03 (0.43 s.e.). This suggests that selection is just as strong, if not stronger, for the group social phenotype, contrary to our *a priori* prediction and to the classic criticism of multilevel selection. Collectively, these results support the importance of a multilevel selection framework to better understand the evolution and maintenance of sociality.

### (b) Antagonistic multilevel selection on sociality

These results suggest not only that multilevel selection may be occurring in the wild for individual social behaviour and group social structure, but also that there is antagonistic multilevel selection *within* each of the two phenotypic levels and *between* the two phenotypic levels [9,33,34]. For adult females, there is both positive and negative selection within the individual (between connectivity and closeness) and within the group social phenotype (between closeness, breakability and clustering). There is also antagonistic selection between the two social phenotypes for adult females: positive selection for more social individuals potentially being counteracted by selection for less social groups, and *vice versa*. Antagonistic multilevel selection *within* and *between* social phenotypes may flatten or constrain the overall selection pressure for both social phenotypes [20,33], which could explain why sociality is not as highly heritable (*h^2^* = 0.11 [42]) or ubiquitously beneficial in this system as it is in other, more gregarious systems [12], such as humans (*Homo sapiens*; *h^2^* = 0.47 [96]) and rhesus macaques (*Macaca mulatta*; *h^2^* = 0.84 [97]).

In a captive population of forked fungus beetles (*Bolitotherus cornutus*) with a fixed social group size, Costello *et al.* [9] showed positive selection for more connected individual social phenotypes in males and negative selection for more connected group social phenotypes in females within the context of reproductive success, suggesting sexually antagonistic multilevel selection (as we also observe, but across different fitness contexts). That study quantified individual and group social phenotypes with an analogous social network trait and contextual analysis approach similar to ours here, but without quantifying the independent contributions of selection for multiple analogous pairs in the same model. Combined with our results, this shows multilevel selection for social phenotypes is present in Coleoptera and Rodentia. This evidence of multilevel selection in the wild and across species provides further impetus for experimental work to manipulate the composition of individual and group social phenotypes to further disentangle these nested, inherently non-independent, structures and to nail down causality and eliminate unmeasured variables [39]. For example, future work should leverage wild populations to identify, and experimental populations to confirm, the presence of multilevel selection and the factors that may mediate the strength and context of multilevel selection. Factors such as relatedness, phenotypic plasticity, individual and group age, group composition and sex ratios, and environmental factors (such as resource distribution) could increase or decrease the strength and direction of multilevel selection in other species. Future work should also consider nonlinear selection, in order to explore disruptive, stabilizing and correlational selection within and between levels of sociality. We did not have sufficient data to appropriately account for nonlinear selection in all models in this current study (for the models that did converge, the biological interpretation of the results was quantitatively similar to the results presented here).

### (c) Multilevel adaptation in response to multilevel selection

Because the contextual analysis regressed individual fitness against the individual and group social phenotypes (i.e. partitioning selection among the two levels relative to each other), our results of selection at both levels add support to the distinct and discrete nature of individual behaviour and group social structure despite being nested levels of biological organization [8–10,13–15,18,20,21,37]. Given we now show selection at multiple phenotypic levels in addition to heritability [42] for social behaviour in this system, this allows the potential for an evolutionary response. Evidence for multilevel adaptation in response to multilevel selection would require demonstration of intergenerational changes in the units of replication that underlie social behaviours [51]. For example, in fruit flies (*Drosophila melanogaster*), the gene *CG14109* influences betweenness centrality [98], a key property of social networks that indicates an individual’s importance in facilitating disease spread, communication and cohesion within the network. Future work should leverage genomic tools to link individual and group phenotypes with candidate genes, like *CG14109,* and to track intergenerational changes in the units of replication that underlie social behaviours.

Given sociality inherently involves interactions between varying phenotypes, indirect genetic effects likely play a role in the evolution of sociality [39,99,100]. Furthermore, in these nested levels of biological organization, selection at one level may accelerate or constrain evolution at another level [39], which is further complicated by the antagonistic multilevel selection within and between social phenotypes we show here. The next steps to better understanding the evolution of societies across species should identify the genetic architecture and correlations underpinning multilevel selection, incorporating both direct and indirect genetic effects in a unified empirical framework [99].

### (d) Group living and social phenotypes

Interestingly, we found no selection for either social phenotype in the context of summer survival. Predation accounts for 98% of summer mortality in this system [79] and the evolutionary origins of yellow-bellied marmot group living partly involve predator avoidance [41]. This suggests selection driving group living can be, and is in some cases, different from the subsequent selection pressures driving how social individuals are and the structure of the social group. Again, work in more gregarious species where the evolutionary origins of group living may be attributable to grooming, heat retention, or resource acquisition could find that the selection for group living and the individual and group social phenotypes are more aligned.

A common argument against group selection (and thus multilevel selection) used since the 1960s focuses on differences in individual lifespan versus group duration [24,52,101]: where individual lifespans are shorter than group duration, selection proceeds faster at the level of the individual. However, in yellow-bellied marmots, dispersal, mortality and births ensure that groups restructure annually. Individuals surviving to adulthood have a mean lifespan of 4.07 years and are thus members of multiple social groups in their lifetime [41]. In this population, individual lifespan is longer than group turnover and thus this argument does not apply. Indeed, the same logic would predict stronger selection for the group phenotype, which we found.

## 4. Conclusion

By leveraging a 19 year dataset from a wild, free-living mammal and contextual analysis to partition selection relatively among two nested levels of sociality, we provide evidence that suggests that multilevel selection is a potentially important and overlooked force in the evolution of sociality. While we have identified multilevel selection, we have not demonstrated evolution in response to this selection, which will require additional work. We recognize that experimental tests are required to formalize causality and eliminate unmeasured variables affecting fitness and social phenotypes beyond what our models controlled for.

Nevertheless, our detection of multilevel selection in the wild provides the impetus to develop these multilevel selection experiments across systems. Considering levels of sociality independently may be reductive and lead to Type I or Type II errors; when we do not incorporate a multilevel selection perspective, we may incorrectly attribute natural selection to the levels of organization not actually under direct natural selection. Instead, sociality can be quantified as a nested, multidimensional phenotype of which different levels can be selected on and change with age and sex. Compared with exploring levels of sociality independently and given contextual analysis partitions selection among phenotypic levels relative to each other, multilevel selection may more comprehensively represent how natural selection acts on the social interactions and structures that arise from group living. We provide empirical evidence to spark further study in this classic debate in biology.

## Data Availability

Data and code to reproduce analyses are available at [102]. Supplementary material is available online [103].
